# Evaluation of the Effectiveness of Peer Education in Improving HIV Knowledge, Attitude, and Sexual Behaviours among In-School Adolescents in Osun State, Nigeria

**DOI:** 10.1155/2014/131756

**Published:** 2014-11-13

**Authors:** Adeleye Abiodun Adeomi, Oluwatosin Adediran Adeoye, Esther Olufunmilayo Asekun-Olarinmoye, Olugbemiga Lanre Abodunrin, Adenike Iyanuoluwa Olugbenga-Bello, Adedayo Olukemi Sabageh

**Affiliations:** ^1^Department of Community Medicine, Ladoke Akintola University of Technology (LAUTECH) Teaching Hospital, PMB 4007, Ogbomoso, Oyo State, Nigeria; ^2^Department of Community Medicine, Osun State University, PMB 4494, Osogbo, Osun State, Nigeria; ^3^Department of Community Medicine, College of Health Sciences, Ladoke Akintola University of Technology (LAUTECH), PMB 4000, Ogbomoso, Oyo State, Nigeria

## Abstract

*Introduction*. Young people are at the centre of the global HIV/AIDS epidemic. This study therefore aimed to evaluate the effectiveness of peer education in improving HIV knowledge, attitude, and preventive practices among in-school adolescents in Osun State, Nigeria.* Methods*. This was an intervention study that was carried out among in-school adolescents attending mixed secondary schools in Osun State, Nigeria. The study was in three stages: before intervention, intervention, and after intervention. The impact of peer education was evaluated twelve weeks after intervention. Data were collected using pretested semistructured questionnaires and data analysis was done with SPSS version 16.* Results*. At the preintervention stage, the study and control groups were similar in their sociodemographic characteristics, HIV knowledge, attitude, and preventive practices, including high risk behaviours for HIV/AIDS transmission. After the peer education intervention, those with good knowledge and positive attitudes towards HIV/AIDS increased significantly from 50.0% to 86.7% and from 49.0% to 85.6%, respectively (*P* < 0.05).* Conclusion*. The study showed that peer education is effective in improving knowledge, attitude, and some preventive practices towards HIV/AIDS among in-school adolescents. Educational programmes about HIV/AIDS should therefore be designed to target this age group putting into consideration their unique characteristics.

## 1. Introduction

Over the past three decades, Human Immunodeficiency Virus (HIV)/Acquired Immune Deficiency Syndrome (AIDS) has emerged as a major health, human, and development crisis, especially in Africa, the epicentre of the global AIDS epidemic [1]. Although much has been achieved in the fight against HIV/AIDS, there is still a long way to go in preventing new infections [2].

Young people are at the centre of the global HIV/AIDS epidemic, in regard to both new infections and opportunities for halting the transmission of HIV [3]. Currently, the highest rates of new infections occur in this age group (i.e., 15–24 years) [4]. Research results also indicate that many young people are still at risk because of high-risk sexual behaviours, despite sound knowledge about sexual health risks. The level of perceived vulnerability in this group has been documented to be low, and unprotected sex was common [5–7]. There is growing evidence from several countries where HIV prevalence is decreasing that it is the young people who are reversing the trends [3], since they are the ones more likely to adopt new behaviours [7].

About one out of every three young people newly infected with HIV in 2009 was from South Africa or Nigeria [8]. The 2008 HIV/Syphilis Sentinel Survey in Nigeria revealed that 3.3% of young people aged 15–19 are infected with the HIV virus [9]. According to UNAIDS, Nigeria has estimated 280,000 adolescents living with HIV/AIDS, consisting of 180,000 females and 100,000 males. The number of new infections among young people in Nigeria aged 15–24 years in 2009 was 120,000, which is nearly 60% of all new infections among young people in West and Central Africa [10, 11].

Peer education has been described as a core pillar of HIV prevention efforts globally [12] and has been found to be effective at improving knowledge and promoting attitudinal and behavioural change [13–16]. Adolescents who believe their friends are not in favour of sexual intercourse for teenagers have been found to be more likely not to engage in sexual intercourse [17], and those who perceive their peers as having a favourable attitude toward condom use are more likely to use a condom themselves [18]. By using peers as resources, information, skills, and caring can be extended in an exponential way and the social climate can be enhanced. Peer education and support can be especially effective among adolescents because friends are their main sources of information about sexual practices, and peer influence often motivates their behavior [7, 19].

Peer education among in-school adolescents is not a new concept; it has been extensively used in places like South Africa [5, 7, 12, 20, 21], but the story in Nigeria is a little different. Research efforts into this area are still relatively little in Nigeria; therefore, there is a need to evaluate the effectiveness of peer education in improving HIV knowledge, attitude, and preventive practices among in-school adolescents in Osun State, Nigeria, with a view to making recommendations that will ultimately help in combating the menace of HIV/AIDS among Nigerian adolescents.

## 2. Materials and Methods

The study area is Osun State which is situated in the Southwestern part of Nigeria and was carved out of old Oyo State in 1991. Osun state has 30 Local Government Areas (LGAs) with one area office. The study population was adolescents (10–19 years) attending selected mixed secondary schools in Osun State, Nigeria.

This was an intervention study (the quasiexperimental type) carried out in three stages, namely, preintervention, intervention, and postintervention stages. At the preintervention stage, the baseline information for respondents in the two groups was obtained. At the intervention stage, recruitment and training of 48 students as peer educators (two in each class for the two schools used as study group) were carried out for two weeks. Topics covered include HIV/AIDS, other sexually transmitted infections, and techniques for sharing information. The training was in form of lectures, motivational talks, and demonstrations using audiovisuals, posters, role plays, and practical demonstration. At the completion of the training, the trained peer educators were provided with educational materials (such as hand bills, leaflets, posters, etc.).

The peer educators, with the support of the teachers, were encouraged to use innovative approaches to address the reproductive health needs and concerns of the students' population, especially as it relates to HIV/AIDS. A coordinator and an assistant were selected from the peer educators in each of the two schools for the study group. Meetings were held weekly by the peer educators in each of the schools where they discussed their progresses and challenges, and this was reported to the researcher by the respective coordinators through the mobile phone or during supervisory visits. The researcher met with the peer educators fortnightly to provide them with supportive supervision. At the postintervention stage, the same questionnaire used in the preintervention stage was administered to the same students who were selected at the preintervention stage in both the study and the control groups 12 weeks after intervention.

The multistage sampling technique was used to select the 400 respondents (200 each for both study and control groups). Two out of the three senatorial districts in Osun State were randomly selected at stage one. In stage two, two LGAs, each from the selected senatorial districts, were randomly selected, making a total of four LGAs. A secondary school was randomly selected in each of the selected LGAs in stage three. In stage four, the desired sample size was selected using stratified random sampling with proportional allocation of respondents from the different classes in the selected secondary schools; stratification was along the line of classes (JSS 1 to SS 3). A total of 23, 30, 35, 36, 35, and 41 respondents were randomly selected from JSS 1 to SS 3, respectively, for the study group. For the control group, 25, 26, 28, 37, 41, and 43 respondents were randomly selected from JSS 1 to SS 3, respectively, making a total of 200 respondents for each group.

The United Nations Population Fund's (UNFPA) training of trainers manual [22] and the United Nations Children Fund's (UNICEF) Reproductive Health and HIV/AIDS prevention project's manual for peer educators, produced for the National Youth Service Corps (NYSC) in Nigeria [23], were adapted for use as the training instrument for the peer educators. The adaptation was done after the preintervention stage, so that the particular needs of the students were put into consideration. A pretested semistructured questionnaire was used as the survey instrument. The questionnaires were self-administered by the students under the supervision of the trained research assistants.

The questions about knowledge and attitude of the respondents about HIV/AIDS were scored. For questions whose responses were either yes or no (or correct and incorrect), a correct answer was scored 1 and a wrong answer was scored 0. For questions with three responses (yes, no, and do not know), the correct response was scored 2, the wrong response was scored 0, and do not know or no idea was scored 1. For the questions about attitude that had strongly agree, agree, indifferent, disagree, and strongly disagree options, the responses were scored 5, 4, 3, 2, and 1 in that order for a positive attitude and 1, 2, 3, 4, and 5 for a negative attitude. The sum of the scores for individual respondents was then calculated, and the mean of all the scores was thereafter calculated. The maximum scores possible for knowledge and attitude were 35 and 45 and the mean scores calculated were 24.4 and 30.0, respectively. The respondents who scored below the mean were regarded as having poor knowledge or negative attitude, while those who scored up to or above the mean were regarded as having good knowledge or positive attitude, as the case may be.

The questionnaires were manually sorted out and entered into a computer and the obtained data were analyzed using Statistical Package for Social Sciences (SPSS) version 16. Level of significance was set with *P* value less than or equal to 0.05.

Ethical clearance for the study was obtained from the Ethical Review Committee of Ladoke Akintola University of Technology (LAUTECH) Teaching Hospital and a major limitation was that concurrent health education of the respondents during the time of the study from other sources such as electronic and print media was unavoidable.

## 3. Results

Two hundred questionnaires were administered to each of the study and control groups at the preintervention stage of the survey, and all the questionnaires were completed, giving a response rate of 100%. At the postintervention stage, however, 195 and 192 questionnaires were administered to the study and control groups, respectively, because of the absence of some of the respondents (attrition rate of 2.5% and 4.0%, resp.). All the questionnaires were completed at this stage also, giving a response rate of 100%.

The study and control groups were properly matched such that there was no statistically significant difference in their sociodemographic characteristics (Table 1). Also, prior to intervention, there was no statistically significant difference in the knowledge (Table 2), attitude (Table 3), and sexual behaviours (Table 4) of the adolescents in the study and control groups.

After the intervention, the level of awareness about AIDS significant increased from 96.5% to 100% in the study group, while there was no significant difference in the control group. Furthermore, significantly more respondents in the study group knew the meaning of AIDS (*P* < 0.001), the causative agent for AIDS (*P* < 0.0001), that an HIV infected person could still look healthy (*P* < 0.0001), and that HIV had no cure (*P* < 0.0001). There was also significant improvement in the knowledge of the respondents in the study group about modes of transmission and prevention of HIV/AIDS, while there was no such significant improvement among the respondents in the control group. After scoring the outcome variables for knowledge about HIV/AIDS, those with good knowledge significantly increased after intervention from 100 (50.0%) to 169 (86.7%) among the study group, but not in the control group (Table 5).

After the scoring and categorization of the respondents attitude in the preintervention stage, 102 (51.0%) and 92 (46.0%) had negative attitude, while 98 (49.0%) and 108 (54.0%) had positive attitudes in the study and control groups, respectively. After the intervention, those with positive attitudes in the study group significantly increased to 167 (85.6%), while there was no significant difference in the control group before and after the intervention.

After intervention, there was a significant reduction in the mean sexual exposures (*P* = 0.02) and the number of sexual partners (*P* = 0.02) among the study group in the 3 months after the intervention. Significantly more of the adolescents in the study group also planned to delay sex until they were married (*P* = 0.03). There was, however, no significant change in the sexual behaviour of the respondents in the control group at the postintervention stage (*P* > 0.05) (Table 6).

## 4. Discussion

The respondents in both study and control groups were similar in their sociodemographic characteristics as evidenced by the fact that there was no statistically significant difference in their age, gender, ethnicity, religion, family settings, parents' occupation, and even their class distribution. Furthermore, there was no statistically significant difference in their knowledge, attitude, and preventive practices about HIV/AIDS at the preintervention stage of the study. This lays an important background for the later evaluation of the intervention programme.

Although the level of awareness about AIDS at the preintervention stage was very high with more than 9 out of 10 respondents in both the study and control groups being aware of the disease called AIDS, the comprehensive knowledge about HIV/AIDS was rather poor. Nearly four-fifths of the respondents did not know the correct meaning of AIDS and more than 9 out of 10 did not know the agent that causes AIDS. About three-fifths of the respondents did not know that a person infected with HIV could still look healthy and half did not know that AIDS has no cure.

In a systematic review conducted by Medley et al. [24] about peer education as an effective behaviour change strategy in developing countries, peer education reportedly had the strongest impact on changing HIV knowledge and attitudes. This finding was corroborated by this study because the intervention had a positive impact on practically every aspect of the students' knowledge about HIV/AIDS. After intervention, all the students in the study group were aware of AIDS, more than 7 out of 10 knew the correct meaning of AIDS, and four-fifths knew that a person infected with HIV could still look healthy. Also, nearly all the respondents in the study group knew blood transfusion, sexual intercourse, and unsterilized sharp objects as modes of transmission of HIV after intervention, with only about a fifth of the respondents mentioning mosquitoes as a mode of transmission of HIV in contrast to the more than 70% at before intervention. Significantly, more of the respondents in the study group knew that AIDS has no cure after intervention, with most knowing that help was available to those infected with the disease. There was, however, no such significant difference in the knowledge about HIV/AIDS before and after intervention among the control group.

Categorized knowledge about HIV/AIDS showed that nearly 9 out of 10 of the respondents in the study group had good comprehensive knowledge after intervention compared to the half who had good knowledge before intervention, and this finding was statistically significant. This was not the case for those in the control group were those who had good knowledge only marginally increased after intervention, without any statistical significance. This finding agrees with the results from previous studies among in-school adolescents that knowledge can increase after educational interventions [24–27], and it is very encouraging. Although knowledge alone is often not sufficient in itself to produce change in sexual behaviour in most people, but acquisition of knowledge is usually the first stage in the process of changes in behavior [25]. Increased comprehensive correct knowledge has also been found to reduce HIV incidence and prevalence in most countries with high HIV prevalence [10].

Before intervention, while about 7 out of 10 were willing to care for a family member with HIV, less than half were willing to eat from the same plate with a person living with HIV/AIDS or sleep in the same room with someone infected with HIV and only 1 out of 5 respondents agreed that they belonged to the at-risk group for HIV infection. The categorized attitude of the respondents after scoring the outcome variables showed that only half of the respondents had a positive attitude towards HIV/AIDS and those living with it. This finding corroborates the findings of previous studies which generally report a negative attitude towards HIV/AIDS and those living with it among adolescents [28–31].

Peer education also had a significant impact on the students' attitudes towards HIV/AIDS and people living with it. The students in the study group had significantly better disposition to almost all the issues raised to assess their attitudes towards HIV/AIDS and people living with it after intervention. More than four-fifths were then willing to sleep in the same room with a person living with HIV/AIDS as opposed to the less than half before intervention, and more than three-fifths agreed that they belonged to the at-risk group for HIV infection as compared with the one-fifth before intervention. Categorized attitude showed that about 4 out of 5 respondents had positive attitudes after intervention as compared with the less than half before intervention. There was, however, no such significant change in the control group. This finding agrees with results from previous studies that peer-led intervention can have a positive effect on the attitude of secondary school students towards reproductive issues like HIV/AIDS [13, 14, 16, 25, 26, 32, 33]. This positive change in attitude is very important and encouraging, because attitude has been said to be a predictor of intentions to undertake any behavior [25, 34].

A little more than 20% of the respondents had had sexual intercourse before. More than a quarter of those that were sexually exposed had sexual intercourse with more than one sexual partner in the three months preceding the study, and more than 1 in 5 of them had paid someone or demanded payment for sex within the same time period. This pattern of high risk sexual behaviour has been well established by previous studies, though the findings vary from place to place [25, 30, 35–45].

There was a significant reduction in the number of sexual partners reported among students in the study group after intervention, while students in the control group had no significant difference before and after intervention; rather the number of those sexually exposed in the control group increased slightly. There was also a significant reduction in the mean number of sexual exposures among students in the study group with significantly more of those not sexually active resolving to wait till marriage before having sex after intervention, but no such significant difference among students in the control group. This compares well with the study by Ajuwon and Brieger [25] who found a decrease in sexual partners after some secondary students in rural Southwestern Nigeria were exposed to peer education. Other researchers have also reported similar findings among secondary school students [7, 26]. This finding is important because reduction in the number of sexual partners lowers the risk of exposure to STIs including HIV/AIDS [25]. This may actually be the reason why there was a significant reduction in the students in the study group reporting symptoms suggestive of STI after intervention, as compared with those in the control group where there was no statistical difference observed.

## 5. Conclusion and Recommendation

The study shows that peer education is effective in improving HIV knowledge, attitude, and some preventive practices among in-school adolescents. It also shows that, if provided with adequate training and supportive supervision, students can be agents of change in the school environment. Adolescents are an important group in the war against HIV/AIDS; it is therefore recommended that educational programmes about HIV/AIDS should be designed to target this age group putting into consideration their unique characteristics. Educational programmes about HIV/AIDS targeted at this age group should consider using peer education for effective outcomes.

## Figures and Tables

**Table 1:** Sociodemographic characteristics of the respondents.

Sociodemographic characteristics	Frequency (percent)			Statistics
	Study (*n* = 200)	Control (*n* = 200)	Total (*n* = 400)	
Age group (in years)				
10–13 (early adolescence)	79 (39.5)	75 (37.5)	154 (38.5)	*χ* ^2^ = 0.26 *P* = 0.88
14–16 (middle adolescence)	92 (46.0)	93 (46.5)	185 (46.2)	
17–19 (late adolescence)	29 (14.5)	32 (16.0)	61 (15.2)	
Gender				
Male	91 (45.5)	103 (51.5)	194 (48.5)	*χ* ^2^ = 1.44 *P* = 0.23
Female	109 (54.5)	97 (48.5)	206 (51.5)	
Ethnicity				
Yoruba	197 (98.5)	199 (99.5)	396 (99.0)	*χ* ^2^ = 1.01 ^*^ *P* = 0.62
Others	3 (1.5)	1 (0.5)	4 (1.0)	
Religion				
Christianity	133 (66.5)	120 (60.0)	253 (63.2)	*χ* ^2^ = 2.36 ^**^ *P* = 0.31
Islam	65 (32.5)	79 (39.5)	144 (36.0)	
Traditionalist	2 (1.0)	1 (0.5)	3 (0.8)	
Family setting				
Monogamous	103 (51.5)	106 (53.0)	209 (52.2)	*χ* ^2^ = 09 *P* = 0.76
Polygamous	97 (48.5)	94 (47.0)	191 (47.8)	
Father's occupation				
Unemployed	20 (10.0)	18 (9.0)	38 (9.5)	*χ* ^2^ = 1.26 *P* = 0.74
Skilled	76 (38.0)	87 (43.5)	163 (40.8)	
Semiskilled	85 (42.5)	78 (39.0)	163 (40.8)	
Unskilled	19 (9.5)	17 (8.5)	36 (9.0)	
Mother's occupation				
Unemployed	11 (5.5)	13 (6.5)	24 (6.0)	*χ* ^2^ = 1.35 *P* = 0.72
Skilled	52 (26.0)	54 (27.0)	106 (26.5)	
Semiskilled	128 (64.0)	128 (64.0)	256 (64.0)	
Unskilled	9 (4.5)	5 (2.5)	14 (3.5)	

^*^Fisher's exact test was used; ^**^likelihood ratio used.

**Table 2:** Preinterventional knowledge of respondents about HIV/AIDS.

Variable	Frequency (percent)			Statistics
	Study	Control	Total	
Awareness about AIDS				
Yes	193 (96.5)	194 (97.0)	387 (96.8)	*χ* ^2^ = 0.08 *P* = 0.78
No	7 (3.5)	6 (3.0)	13 (3.2)	
Meaning of AIDS				
Correct	38 (19.7)	48 (24.7)	86 (22.2)	*χ* ^2^ = 1.43 *P* = 0.23
Incorrect	155 (80.3)	146 (75.3)	301 (77.8)	
Causative agent for AIDS				
Correct (HIV)	15 (7.8)	18 (9.3)	33 (8.5)	*χ* ^2^ = 0.28 *P* = 0.60
Incorrect	178 (92.2)	176 (90.7)	354 (91.5)	
HIV infected person can still look healthy				
Yes	92 (46.0)	85 (42.5)	177 (44.2)	*χ* ^2^ = 2.41 *P* = 0.30
No	100 (50.0)	100 (50.0)	200 (50.0)	
Do not know	8 (4.0)	15 (7.5)	23 (5.8)	
HIV/AIDS can be cured				
Yes	75 (37.5)	77 (38.5)	152 (38.0)	*χ* ^2^ = 1.07 *P* = 0.59
No	103 (51.5)	95 (47.5)	198 (49.5)	
Do not know	22 (11.0)	28 (14.0)	50 (12.5)	
Modes of transmission of HIV				
(Multiple response)				
Sexual intercourse	184 (92.0)	182 (91.0)	366 (91.5)	*P* = 0.86
Transfusion of unscreened blood	186 (93.0)	176 (88.0)	362 (90.5)	*P* = 0.11
Unsterilized sharp objects	180 (90.0)	178 (89.0)	358 (89.5)	*P* = 0.63
Unsterilized surgical instruments	170 (85.0)	170 (85.0)	340 (85.0)	*P* = 0.97
Mother to child during pregnancy	166 (83.0)	151 (75.5)	317 (79.2)	*P* = 0.17
Breast milk	156 (78.0)	151 (75.5)	307 (76.8)	*P* = 0.67
Mosquitoes	143 (71.5)	143 (71.5)	286 (71.5)	*P* = 0.95
Coughing and sneezing	84 (42.0)	83 (41.5)	167 (41.8)	*P* = 0.99
Spiritual means	64 (32.0)	70 (35.0)	134 (33.5)	*P* = 0.70
Casual contact	28 (14.0)	32 (16.0)	60 (15.0)	*P* = 0.38
Modes of prevention of HIV/AIDS				
(Multiple response)				
Not sharing sharp objects	154 (77.0)	162 (81.0)	316 (79.0)	*P* = 0.61
Condom	139 (69.5)	146 (73.0)	285 (71.2)	*P* = 0.62
Faithfulness to a partner	121 (60.5)	136 (68.0)	257 (64.2)	*P* = 0.29
Abstinence	107 (53.5)	115 (57.5)	222 (55.5)	*P* = 0.68
Knowledge categories				
Poor knowledge	100 (50.0)	102 (51.0)	202 (50.5)	*χ* ^2^ = 0.04 *P* = 0.84
Good knowledge	100 (50.0)	98 (49.0)	198 (49.5)	

**Table 3:** Attitude of respondents towards HIV/AIDS at the preintervention stage.

Variables	Study (%) (*n* = 200)			Control (%) (*n* = 200)			*P* value
	Agree	Do not know	Disagree	Agree	Do not know	Disagree	
HIV exists in Nigeria	184 (92.0)	5 (2.5)	11 (5.5)	193 (96.5)	4 (2.0)	3 (1.5)	0.08
Willing to care for a family member with HIV	146 (73.0)	10 (5.0)	44 (22.0)	142 (71.0)	16 (8.0)	42 (21.0)	0.48
Safe to buy fresh vegetables from a shopkeeper with HIV	110 (55.0)	12 (6.0)	78 (39.0)	109 (54.5)	16 (8.0)	75 (37.5)	0.73
Allow teacher with HIV to continue teaching in class	122 (61.0)	13 (6.5)	65 (32.5)	129 (64.5)	12 (6.0)	59 (29.5)	0.77
Willing to eat from the same plate with PLWHA	99 (49.5)	8 (4.0)	93 (46.5)	95 (47.5)	11 (5.5)	94 (47.0)	0.76
Willing to sleep in the same room with someone infected with HIV	97 (48.5)	17 (8.5)	86 (43.0)	106 (53.0)	16 (8.0)	78 (39.0)	0.66
Risky to shake hands with or hug a person living with HIV/AIDS	108 (54.0)	10 (5.0)	82 (41.0)	86 (43.0)	11 (5.5)	103 (51.5)	0.09
Continue my friendship with a friend infected with HIV	111 (55.5)	15 (7.5)	74 (37.0)	121 (60.5)	19 (9.5)	60 (30.0)	0.31
I belong to the at-risk group for HIV infection.	39 (19.5)	14 (7.0)	147 (73.5)	41 (20.5)	15 (7.5)	144 (72.0)	0.94

**Table 4:** Respondents' sexual exposure at the preintervention stage.

Variables	Frequency (percent)			Statistics
	Study	Control	Total	
Ever had sexual intercourse				
Yes	51 (25.5)	39 (19.5)	90 (22.5)	*χ* ^2^ = 2.07 *P* = 0.15
No	149 (74.5)	161 (80.5)	310 (77.5)	
Total	**200 (100.0)**	**200 (100.0)**	**400 (100.0)**	

Mean age at first sex (in years)	12.5 ± 2.5 *n* = 51	13.0 ± 3.0 *n* = 39	12.7 ± 2.7 *n* = 90	*P* = 0.34

Nature of first sexual intercourse				
Planned	25 (49.0)	17 (43.6)	42 (46.7)	*χ* ^2^ = 4.02 *P* = 0.13
Casual	16 (31.4)	19 (48.7)	35 (38.9)	
Forced	10 (19.6)	3 (7.7)	13 (14.4)	
Total	**51 (100.0)**	**39 (100.0)**	**90 (100.0)**	

Mean number of sexual exposures in the last 3 months	1.6 ± 1.5 *n* = 51	1.9 ± 2.1 *n* = 39	1.7 ± 1.8 *n* = 90	*P* = 0.42

Sexual partners in the last 3 months				
Nil	13 (25.5)	10 (25.6)	23 (25.6)	*χ* ^2^ = 0.36 *P* = 0.84
1	25 (49.0)	17 (43.6)	42 (46.7)	
2 or more	13 (25.5)	12 (30.8)	25 (27.8)	
Total	**51 (100.0)**	**39 (100.0)**	**90 (100.0)**	

Mean number of sexual partners in the last 3 months	1.2 ± 1.3 *n* = 51	1.3 ± 1.4 *n* = 39	1.2 ± 1.3 *n* = 90	*P* = 0.83

Paid sex in the last 3 months				
Yes	11 (21.6)	9 (23.1)	20 (22.2)	*χ* ^2^ = 0.21 *P* = 0.87
No	40 (78.4)	30 (76.9)	70 (77.8)	
Total	**51 (100.0)**	**39 (100.0)**	**90 (100.0)**	
Plan for sex for those who had not had sex before				
When I am married	121 (81.2)	130 (80.7)	251 (81.0)	*χ* ^2^ = 7.34 ^*^ *P* = 0.20
When I finish secondary school	11 (7.4)	11 (6.8)	22 (7.1)	
Whenever there is opportunity	13 (8.7)	7 (4.3)	20 (6.5)	
Whenever I feel like it	2 (1.3)	6 (3.7)	8 (2.6)	
When I have a boy- or girlfriend	2 (1.3)	4 (2.5)	6 (1.9)	
When I am a little older	0 (0.0)	3 (1.9)	3 (1.0)	
Total	**149 (100.0)**	**161 (100.0)**	**310 (100.0)**	

^*^Likelihood ratio used.

**Table 5:** Postinterventional knowledge of respondents about HIV/AIDS.

Variable	Study (percent)			Control (percent)		
	Pre (*n* = 200)	Post (*n* = 195)	*P* value	Pre (*n* = 200)	Post (*n* = 192)	*P* value
Awareness about AIDS						
Yes	193 (96.5)	195 (100.0)	0.01	194 (97.0)	186 (96.9)	0.94
No	7 (3.5)	0 (0.0)		6 (3.0)	6 (3.1)	
Meaning of AIDS						
Correct	38 (19.7)	141 (72.3)	<0.001	48 (24.7)	47 (24.5)	0.91
Incorrect	155 (80.3)	54 (27.7)		146 (75.3)	145 (75.5)	
Causative agent for AIDS						
Correct (HIV)	15 (7.8)	127 (65.1)	<0.001	18 (9.3)	20 (10.4)	0.64
Incorrect	178 (92.2)	68 (34.9)		176 (90.7)	172 (89.6)	
HIV infected person can still look healthy						
Yes	92 (46.0)	154 (79.0)	<0.001	85 (42.5)	84 (43.8)	0.51
No	100 (50.0)	32 (16.4)		100 (50.0)	99 (51.6)	
Do not know	8 (4.0)	9 (4.6)		15 (7.5)	9 (4.7)	
HIV/AIDS can be cured						
Yes	75 (37.5)	43 (22.1)	<0.001	77 (38.5)	83 (43.2)	0.45
No	103 (51.5)	143 (73.3)		95 (47.5)	89 (46.4)	
Do not know	22 (11.0)	9 (4.6)		28 (14.0)	20 (10.4)	
Modes of transmission of HIV						
(Multiple response)						
Sexual intercourse	184 (92.0)	194 (99.5)	<0.001	182 (91.0)	178 (92.7)	0.82
Transfusion of unscreened blood	186 (93.0)	192 (98.5)	0.02	176 (88.0)	173 (90.1)	0.71
Unsterilized sharp objects	180 (90.0)	188 (96.4)	0.03	178 (89.0)	159 (82.8)	0.24
Unsterilized surgical instruments	170 (85.0)	181 (92.8)	0.046	170 (85.0)	176 (91.7)	0.43
Mother to child during pregnancy	166 (83.0)	171 (87.7)	0.01	151 (75.5)	152 (79.2)	0.65
Breast milk	156 (78.0)	171 (87.7)	0.04	151 (75.5)	141 (73.4)	0.76
Mosquitoes	143 (71.5)	36 (18.5)	0.001	143 (71.5)	145 (75.5)	0.67
Coughing and sneezing	84 (42.0)	40 (20.5)	<0.001	83 (41.5)	74 (38.5)	0.64
Spiritual means	64 (32.0)	23 (11.8)	<0.001	70 (35.0)	61 (31.8)	0.78
Casual contact	28 (14.0)	13 (6.7)	0.001	32 (16.0)	27 (14.1)	0.62
Modes of prevention of HIV/AIDS						
(Multiple response)						
Not sharing sharp objects	154 (77.0)	174 (89.2)	0.002	162 (81.0)	152 (79.2)	0.10
Condom	139 (69.5)	169 (86.7)	0.0001	146 (73.0)	145 (75.5)	0.66
Faithfulness to a partner	121 (60.5)	138 (70.8)	0.03	136 (68.0)	130 (67.7)	0.63
Abstinence	107 (53.5)	145 (74.4)	0.0001	115 (57.5)	102 (53.1)	0.68
Knowledge categories						
Poor knowledge	100 (50.0)	26 (13.3)	<0.0001	102 (51.0)	93 (48.4)	0.61
Good knowledge	100 (50.0)	169 (86.7)		98 (49.0)	99 (51.6)	

**Table 6:** Respondents' sexual exposure at pre- and postintervention stages.

Variables	Study group (%)		Control group (%)	
	Pre	Post	Pre	Post
Mean number of sexual exposures in the last 3 months	1.6 ± 1.5	0.9 ± 1.2	1.9 ± 2.1	1.5 ± 1.2
Total	**51 (100.0)**	**49 (100.0)**	**39 (100.0)**	**40 (100.0)**

Statistics	*t* = 2.37; *P* = 0.02		*t* = 1.03; *P* = 0.31	

Sexual partners in the last 3months				
Nil	13 (25.5)	26 (53.1)	10 (25.6)	10 (25.0)
1	25 (49.0)	15 (30.6)	17 (43.6)	19 (47.5)
2 or more	13 (25.5)	8 (16.3)	12 (30.8)	11 (27.5)
Total	**51 (100.0)**	**49 (100.0)**	**39 (100.0)**	**40 (100.0)**

Statistics	*χ* ^2^ = 7.99; df = 2; *P* = 0.02		*χ* ^2^ = 0.14; df = 2; *P* = 0.93	

Mean number of sexual partners in the last 3 months	1.2 ± 1.3	0.7 ± 1.3	1.3 ± 1.4	1.1 ± 0.8
Total	**51 (100.0)**	**49 (100.0)**	**39 (100.0)**	**40 (100.0)**

Statistics	*t* = 1.60; *P* = 0.11		*t* = 0.71; *P* = 0.48	

Paid sex in the last 3 months				
Yes	11 (21.6)	6 (12.2)	9 (23.1)	9 (22.5)
No	40 (78.4)	43 (87.8)	30 (76.9)	31 (77.5)
Total	**51 (100.0)**	**49 (100.0)**	**39 (100.0)**	**40 (100.0)**

Statistics	*χ* ^2^ = 1.54; df = 1; *P* = 0.21		*χ* ^2^ = 0.01; df = 1; *P* = 0.93	

Plan for sex for those who had not had sex before				
When I am married	121 (81.2)	135 (92.5)	130 (80.7)	126 (82.9)
After secondary school	11 (7.4)	5 (3.4)	11 (6.8)	8 (5.3)
When there is opportunity	13 (8.7)	4 (2.7)	7 (4.3)	10 (6.6)
When I feel like it	2 (1.3)	2 (1.4)	6 (3.7)	6 (4.0)
When I have a boy/girlfriend	2 (1.3)	0 (0.0)	4 (2.5)	1 (0.7)
When I am a little older	0 (0.0)	0 (0.0)	3 (1.9)	1 (0.7)
Total	**149 (100.0)**	**146 (100.0)**	**161 (100.0)**	**152 (100.0)**

Statistics	^*^ *χ* ^2^ = 9.75; df = 5; *P* = 0.03		^*^ *χ* ^2^ = 3.58; df = 5; *P* = 0.59	

^*^Likelihood ratio used.
